# Impact of Platelet-Rich Plasma on Viability and Proliferation in Wound Healing Processes after External Radiation

**DOI:** 10.3390/ijms18081819

**Published:** 2017-08-22

**Authors:** Yvonne Reinders, Oliver Felthaus, Gero Brockhoff, Fabian Pohl, Norbert Ahrens, Lukas Prantl, Frank Haubner

**Affiliations:** 1 Department of Otorhinolaryngology, Division of Facial Plastic Surgery, University Hospital Regensburg, 93053 Regensburg, Germany; 2 Department of Plastic, Aesthetic, Hand & Reconstructive Surgery, University Hospital Regensburg, 93053 Regensburg, Germany; oliver.felthaus@ukr.de (O.F.); lukas.prantl@ukr.de (L.P.); 3 Department of Gynaecology and Obstetrics, University Medical Centre Regensburg, 93053 Regensburg, Germany; gero.brockhoff@ukr.de; 4 Department of Radiotherapy, University Hospital Regensburg, 93053 Regensburg, Germany; fabian.pohl@ukr.de; 5 Department of Clinical Chemistry and Laboratory Medicine, University Hospital Regensburg, 93053 Regensburg, Germany; norbert.ahrens@ukr.de; 6 Department of Otorhinolaryngology, University Hospital Goettingen, 37075 Goettingen, Germany; 7 Department of Otorhinolaryngology, Ludwig-Maximilians-University Munich, 81377 Munich, Germany

**Keywords:** radiation therapy, human adipose-derived stem cells, microvascular endothelial cells, platelet-rich plasma

## Abstract

Platelet-rich plasma is a current subject of studies on chronic wound healing therapy due to possible pro-angiogenic effects. Microvascular compromise represents the major component in radiogenic wound healing complications. The effects of platelet-rich plasma on irradiated cells of the cutaneous wound healing process are poorly understood so far. In this study, the interaction of endothelial cells and adipose-derived stem cells in conjunction with treatment with platelet-rich plasma is investigated in the context of radiation effects. Therefore, the expression of surface-marker CD90 and CD31 was determined. Moreover, cell proliferation and viability after external radiation was analyzed with and without treatment by platelet-rich plasma.

## 1. Introduction

Chronic wounds after radiotherapy pose major challenges to head and neck surgery [1]. The malfunction of wound healing in irradiated tissues is associated with fibrosis and decreased vascularity [1,2]. Generally, wound repair comprises three major phases: inflammation, new tissue formation (including cell proliferation, cell migration, neoangiogenesis), and tissue remodeling. Wound healing processes are based on a complex interaction network of cells and extracellular messenger molecules [3]. External radiation provokes serious damage to this well-organized network. Moreover, decline in microvascularization represents a major component in radiogenic wound healing complications. Previously, our group detected a reduction in the cell number of irradiated human dermal microvascular endothelial cells (HDMEC), adipose-derived stem cells (ASC) and the respective co-culture correlating with increasing radiation intensity [2]. Mesenchymal stem cells are suggested to support wound healing due to their production of multiple growth factors and cytokines which are of major interest in wound healing processes [4]. The discovery of adipose-derived stem cells (ASC) also as multipotent progenitors of various cell types was an important step forward in the field of stem cell research [5,6]. Furthermore, ASC can be obtained easily by rather low invasive procedures such as liposuction. According to reports in the area of breast reconstruction, the influence of ASC on chronic wounds seems promising [7,8]. Nevertheless, larger clinical studies of patients with impaired wound healing in the head and neck area which focus on the cellular interactions are still missing. Currently, platelet-rich plasma (PRP) products are suggested in the treatment of chronic wounds due to possible pro-angiogenic effects [9,10,11]. The molecular effects of PRP on irradiated cells in wound healing processes are still poorly understood. During wound healing, platelets are activated and exposed to the bloodstream after endothelial injury. Thereby, platelets secrete stored intercellular mediators and cytokines from the cytoplasm and release their α-granule content after activation for wound healing initiation [12].

Combining the wound healing–promoting attributes of PRP and ASC seems highly promising. Therefore, in vitro studies of the effect on the proliferation of PRP on mono-cultures and co-cultures of HDMEC and ASC are needed. Hence, we evaluated the effect of external radiation on human dermal microvascular endothelial cells and the effects of adipose-derived stem cells in a co-culture setting with or without PRP treatment.

## 2. Results

### 2.1. Effect of Irradiation on Specific Surface Markers Using Flow Cytometry of Mono- and Co-Cultured Cells

**Figure 1:**
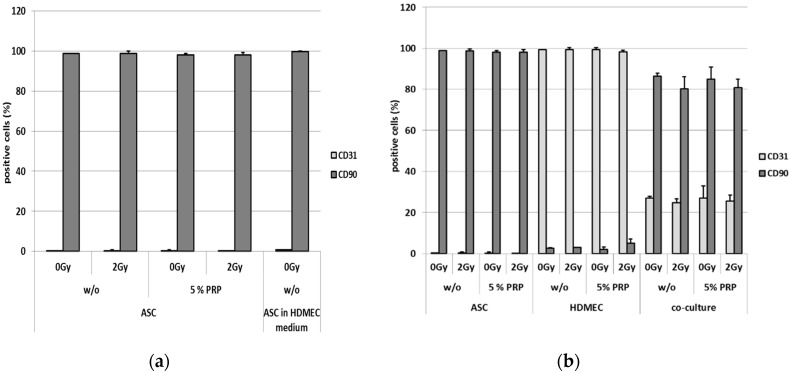
Flow cytometric analysis of the surface markers CD90 and CD31. CD31 is highlighted in light grey, CD90 in dark grey. Samples were treated with 5% platelet-rich plasma and irradiated using a dose of 2 Gy (J/kg). Data for control cells are given. (**a**) Comparison of adipose-derived stem cells in α-MEM and adipose-derived stem cells in EC-Medium; (**b**) Analysis of CD90 and CD31 of adipose-derived stem cells, microvascular endothelial cells and the respective co-culture.

Cells were phenotyped in vitro by a specific panel of markers. In this context, the expression of CD90 as a surface marker for adipose-derived stem cells was determined using flow cytometry. Furthermore, CD31 was used as a surface marker for microvascular endothelial cells. Thereby, ASC were nearly 100% positive for CD90 but negative for CD31 (Figure 1a). Treatment with radiation or application of 5% PRP had no significant effect on the expression of these markers on ASC. Additionally, changing the medium from α-MEM to EC-Medium had no effect on the expression of these surface markers on ASCs. In contrast, HDMEC were nearly 100% positive for CD31 but negative for CD90 (Figure 1b). Again, neither irradiation nor treatment with PRP had any effect on the expression of the surface markers.

All co-cultures consisting of ASC and HDMEC (mixing rate ASC/HDMEC 1:4) showed rates of about one-quarter CD31^+^ cells and slightly more than three-quarters CD90^+^ cells. Hence, treatment with PRP on co-cultured cells as well as treatment with external radiation did not affect the expression of these surface markers on both ASC and HDMEC. However, despite the decreased mixing ratio of 1:4, in comparison to a 1:2 ratio that we used in previous studies, a converted mixing ratio was observed after 3 days of co-cultivation. Therefore, HDMEC presumably show a diminished growth rate in co-culture as seen by the low proportion of CD31^+^ cells. This decrease could be due to the direct interaction with ASC or an alteration in secreted factors such as cytokines, chemokines or growth factors.

### 2.2. Effect of Irradiation on Cell Viability

**Figure 2:**
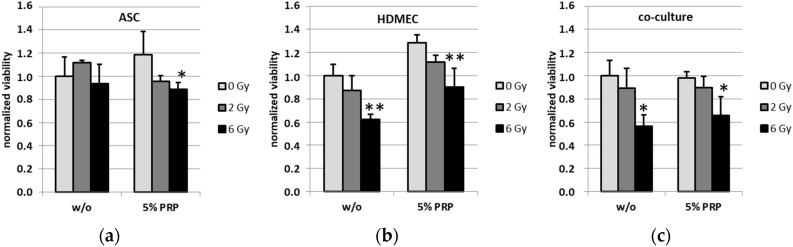
Cell viability determined from adipose-derived stem cells. (**a**) Human dermal endothelial microvascular cells; (**b**) and co-cultures of the respective cell lines; (**c**) upon external radiation and treatment with platelet-rich plasma using a WST-8 assay. Values are represented as means ± standard deviations. Statistical significances of * *p* ≤ 0.05, ** *p* ≤ 0.01 and *** *p* ≤ 0.001 are indicated, respectively.

To analyze the effect of PRP treatment on the cell viability of ASC, HDMEC and the corresponding co-culture upon irradiation, the viability was determined using a colorimetric WST-8 assay. Human ASC showed no altered viability upon radiation (Figure 2a). The treatment of ASC with 5% PRP caused a slight, although not significant, trend towards increased viability which was reversed by irradiation with both tested doses of 2 Gy and 6 Gy, respectively. Endothelial cells showed a trend towards decreased viability upon external radiation, both in the presence and absence of PRP, while a converse trend was observed upon PRP treatment alone (Figure 2b). Moreover, analysis of co-cultured adipose-derived stem cells and endothelial cells showed a significant effect for radiation with 6 Gy in both PRP-treated and untreated cells (Figure 2c).

### 2.3. Effect of Irradiation on Cell Proliferation

**Figure 3:**
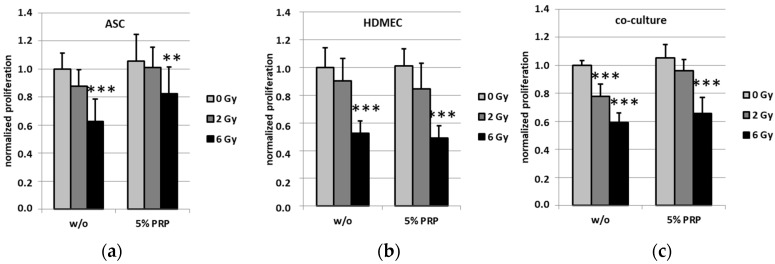
Cell proliferation of ASC, HDMEC and the corresponding co-culture upon treatment with PRP was determined using a BrdU assay. Error bars represent standard deviation. Values are represented as means ± standard deviations. Statistical significances of * *p* ≤ 0.05, ** *p* ≤ 0.01 and *** *p* ≤ 0.001 are indicated, respectively.

The effect of PRP treatment on cell proliferation of ASC, HDMEC and the corresponding co-culture upon irradiation was studied using a colorimetric BrdU assay. All cell cultures showed a trend towards decreasing proliferation after irradiation irrespective of PRP (Figure 3). The proliferation of all cells was significantly diminished by radiation with 6 Gy. Remarkably, untreated co-cultured cells showed a significantly reduced proliferation rate after irradiation with 2 Gy, whereas PRP-treated co-cultures did not. Here, the PRP presence in cell medium had a pro-proliferating effect on cells after radiation.

## 3. Discussion and Conclusions

Compromised wound healing represents a major issue in wound management, particularly after radiation therapy [1,13]. Therefore, methods for improving regeneration and enhancing wound healing are increasingly discussed as a starting point for novel therapies. Platelet-rich plasma as a therapeutic agent for chronic wounds has been a focus in recent years [10,14]. In general, PRP yields pro-proliferative properties for various cell types. It has been shown to contain a variety of essential growth factors, including platelet-derived growth factor (PDGF), vascular endothelial growth factor (VEGF), transforming growth factor-β 1 (TGF-β), and insulin growth factor (IGF) which facilitate repair mechanisms and wound healing [15,16]. These mediators have two primary effects on wound healing: recruiting and activating cells that affect wound healing and the regulation of angiogenesis [17].

Consistently, we report that human adipose-derived stem cells show a slightly enhanced proliferation rate upon treatment with PRP compared to untreated cells, which is consistent with the increased cell count which we previously reported on [9], although the gain in proliferation may be slightly lower possibly due to contact inhibition as the cells may almost reach confluence after incubation.

As impaired microcirculation and inflammation play important roles in compromised wound healing, the concept of therapeutic angiogenesis has become more relevant recently. Thereby, adipose-derived stem cells represent a cell type which is able to migrate and differentiate into endothelial cells. Furthermore, ASC are suggested to support angiogenesis and optimize tissue microcirculation [18,19].

However, emerging evidence suggests that stem cells are also involved in tumor angiogenesis [20]. Thus, a possibly increased risk of tumor recurrence after stem cell therapy has to be an important consideration. The aim of the present study was focused on the possible supportive effects of ASC in co-culture with endothelial cells. Thus, human dermal microvascular endothelial cells were integrated into the co-culture experiments because of their fundamental role in cutaneous wound healing. Endothelial cells display also a high sensitivity to radiation injury. Therefore, cellular events associated with impact on an impaired function of endothelial cells are of particular clinical interest.

Recently, the combination of ASCs and PRP has been reported to yield several beneficial effects, e.g., in bone regeneration [21] and fat graft survival [22]. Particularly in wound management in pathological situations, combination therapy on ASC/PRP was suggested [6,23,24]. To elucidate the cellular processes after radiation of this combination treatment, we focused our experiments of PRP-treated and untreated ASC in co-culture with endothelial cells.

We monitored a potential mixing effect of irradiation and PRP treatment. Surface markers of mono-cultures of HDMEC as well as ASC showed no effect upon radiation or PRP treatment. Although we expected an enhanced ASC growth compared to HDMEC, we applied a mixing ratio of 1:4 (ASC/HDMEC). A three- to four-fold excess of ASC was obtained after 3 days of culture. However, the surface markers of cells in the co-culture were also neither affected upon irradiation nor PRP treatment.

To elucidate the cellular processes after radiation, we investigated differences in proliferation and viability. Irradiation of HDMEC mono-cultures resulted in a significant decrease of proliferation as well as viability, while ASC mono-cultures were less affected by irradiation. This radiation effect was modulated in the co-culture setting with ASC. While the irradiation effects on viability in the co-culture were not affected by PRP treatment, the significant drop in the proliferation rate at a radiation dose of 2 Gy was impeded in PRP-treated co-cultures. Excessive irradiation such as 6 Gy, however, still substantially decreased proliferation of the co-cultures, showing the limits of PRP-exerted protection. With respect to the data of the present study, co-cultured ASC in addition to treatment with PRP seem to protect and/or stimulate HDMEC proliferation. This is in line with our previously published data concerning no PRP-mediated effect on HDMEC but a slight effect on ASC cell count and an even more pronounced effect on ASC/HDMEC co-culture [9]. Taken together, we conclude that the increased co-culture cell counts are indeed a consequence of the pro-proliferative effect exerted by PRP. When ASC and PRP are used in combination therapy, a pro-survival effect may be due to the release of anti-inflammatory chemokines which help reduce inflammation. Hypotheses also exist which suggest PRP may provide nutrient support to the cells through its plasma component and that the fibrin component allows the formation of a scaffold for cells [25,26]. However, the intracellular mechanisms in the endothelial cells that facilitate the protective effects exerted by PRP treatment remain to be elucidated. As stated above, platelets are known to contain various cytokines and growth factors which may activate several promitogenic pathways in the target cells. Moreover, PRP may additionally lead to decreased programmed cell death (PCD), foremost caspase-dependent and -independent apoptosis, although a multitude of other PCD mechanisms such as necroptosis, ferroptosis, pyroptosis or parthanatos could play a minor role as well. The pro-proliferative effects caused by PRP might also be facilitated by interference with, for instance, phosphorylation-dependent signaling cascades regulating mitotic checkpoints. There is a multitude of possible targets such as ATM, p53, CHK1 and 2, CDK1 and 2, AKT, PI3K and CDCs. However, these pathways are interconnected with various other signal transduction pathways regulating important cellular processes such as apoptosis and senescence induction, classical NF-κB-pathway-activation and DNA double-strand and nucleotide excision repair. Therefore, one has to be cautious about the possible deleterious or even pro-tumorigenic side effects of PRP treatment which have to be addressed in future studies. In summary, we found both deleterious effects of radiation as well as the protective effect of PRP. In particular, PRP treatment restores the irradiation-induced proliferation defects of HDMEC when co-cultured with ASC. Moreover, we report a PRP-mediated protective effect on endothelial and adipose stem cell proliferation following radiation. Therefore, a combination of treatment with ASC and PRP products might be useful in the care management of chronic radiogenic wounds. Nevertheless, due to its potential tumor-supporting properties this treatment strategy requires mechanistic exploration in more detail [20].

## 4. Materials and Methods

### 4.1. Cell Culture of HDMEC, ASC and the Respective Co-Culture

Human dermal microvascular endothelial cells (HDMEC, adult donor, catalog number C-12212; PromoCell, Heidelberg, Germany) were maintained in endothelial cell growth medium MV (EC medium, PromoCell, Heidelberg, Germany), the culture incubator was set at 37 °C with 5% carbon dioxide. Cells were used for experiments at passages 3–6.

Adipose-derived stem cells (ASC, isolated as described previously by Gehmert et al.) were maintained in ASC medium (α-MEM containing 10% FBS, 2 mM l-glutamine, and 1% penicillin/streptomycin, Sigma, St. Louis, MO, USA) and were used for experiments at passages 3–6. Briefly, subcutaneous fat tissue—obtained from patients undergoing elective body-contouring procedures—was washed in phosphate-buffered saline, and minced into pieces of <2 mm^3^. Serum-free MEM (1 mL/g tissue) and LiberaseBlendzyme 3 (Roche Diagnostics, Basel, Switzerland) (2 U/g tissue) were added and incubated under continuous shaking at 37 °C for 45 min. The digested tissue was sequentially filtered through 100 μm and 40 μm filters (Fisher Scientific, Schwerte, Germany) and centrifuged at 450× *g* for 10 min. The supernatant was discarded and pelleted cells were washed twice with Hanks’ balanced salt solution (Cellgro, Manassas, VA, USA). Plastic-adherent passage 0 cells were then grown in culture vials (Greiner Bio-one, Frickenhausen, Germany) followed by daily washes to remove red blood cells and non-attached cells. After cells reached 80% confluence in passage 0, they were seeded at a density of 3,000 cells/cm^2^. The culture incubator was set at 37 °C with 5% carbon dioxide. According to the literature ASC remain their differentiation capacity up to passage 15. ASC cultures were harvested and molecular characterized from the Applied Stem Cell Research Center of the University of Regensburg. ASC isolation was in accordance with guidelines of the Declaration of Helsinki for biomedical research. Written consent was obtained from the Institutional Review Board (IRB) of the University of Regensburg to harvest ASC from patients (“Human mesenchymal stem cells as a target for development of cell based regenerative therapies”, IRB #08/117).

For direct co-culture cells of each, HDMEC and ASC, were mixed (mixing rate ASC/HDMEC 1:4) and seeded cell culture flasks. Cells were supplemented with endothelial cell growth medium and treated like mono-cultured cells then.

### 4.2. PRP Preparation

Leukodepleted platelet-rich plasma (PRP) was collected by apheresis from healthy donors using citrate dextrosesolution A as anticoagulant (ACD-A, final concentration 17–20%). Platelet count was determined using an automatic cell-counting system (Sysmex XE-5000, Hyogo, Japan). PRP with at least 10^9^ platelets/L plasma was mixed with an equal volume of thrombin solution (5 U/mL in 40 mM CaCl_2_ buffer, Baxter, Munich, Germany) and was activated for 1 h. Following activation, PRP was centrifuged for 5 min at 4000× *g* to remove cellular debris. The supernatant, containing the released growth factors and cytokines, was sterile filtered through a 0.2 µm filter and stored in aliquots at −20 °C. Activated PRP was used for cell culture experiments at a final concentration of 5% in the culture medium.

### 4.3. External Radiation

A 2 cm perspexplate was positioned above and below the tissue culture plates or flasks to compensate for the buildup effect. Irradiation was delivered via an anterior portal by a 6 MV linear accelerator (3 Gy per min; Synergy, Elekta, Stockholm, Sweden) at room temperature, as previously described by Pohl et al. [27]. The focus–isocenter distance was 100 cm. The isocenter was in the center of the cell medium. Dosimetric evaluations were performed to guarantee a homogenous dose distribution. The cells were irradiated with doses of 2 (0.7 min irradiation) and 6 Gy (2 min irradiation), respectively. Non-irradiated cells under same conditions served as a control.

### 4.4. Cell Proliferation Assay and Vitality Assay

Influence of external radiation on cell proliferation was analyzed using the colorimetric BrdU (5-bromo-2′-deoxyuridine) Cell Proliferation ELISA from Roche (Basel, the Switzerland). Upon addition to cell culture medium BrdU is constantly incorporated into newly synthesized DNA and its amount is proportional to the amount of newly formed DNA and allows comparison of proliferation rates of cells. Briefly, 5000 cells per chamber were seeded as monocultures in a 96-well plate. For co-cultures, 5000 cells (1:4, ASC/HDMEC) were used. Then 24 h after seeding, cells were irradiated with doses of 2 or 6 Gy. Non-irradiated cells of each condition served as controls. After radiation, medium was exchanged. After 48 h BrdU was supplemented to media and cells were incubated for another 24 h. Afterwards, cells were processed following the manufacturer’s instructions, including use of stop solution, and absorption was measured at 450 nm.

Influence of external radiation on cell viability was analyzed using the colorimetric WST-8 Cell Viability kit from Promokine (PromoCell GmbH, Heidelberg, Germany). The tetrazolium salt WST-8 is reduced to water-soluble, orange formazan dye by dehydrogenases present in viable cells. The absorbance of the formazan dye is proportional to the number of metabolically active cells. Briefly, 5000 cells per chamber were seeded as monocultures in a 96-well plate. For co-cultures, 5000 cells (1:4, ASC/HDMEC) were used. Then 24 h after seeding, cells were irradiated with doses of 2 or 6 Gy, respectively. Non-irradiated cells of each condition served as controls. Following radiation, medium was exchanged. After 48 h WST-8 was supplemented to media and cells were incubated for another 24 h. Afterwards, cells were processed following the manufacturer’s instructions and absorption was measured at 450 nm.

### 4.5. Flow Cytometry

Cells were washed 48 h after irradiation with PBS (phosphate-buffered saline, PAALaboratories, Pasching, Austria) and detached by incubation with 500 µL Trypsin/EDTA (Promo-Cell; catalog number C-41000) for 5 min at 37 °C. After neutralization using 1 mL of trypsin neutralizing solution (Promo-Cell; catalog number C-4110, Heidelberg, Germany) cell number was determined at the Cedex XS Analyzer (Innovatis AG, Basel, Switzerland). Cells were counted and 500,000 cells were distributed to each FACS tube. After centrifuging (300× *g*, 5 min) the supernatant was removed and the cells were resuspended in 40 µL staining buffer consisting of PBS containing 0.01% sodium azide, 0.5% BSA, and 2 nM EDTA. Then 5 µL of each antibody (APC anti-human CD90 Antibody, Brilliant Violet 421™ anti-human CD31 Antibody, 100 µg/mL, San Diego, CA, USA) or 5 µL of each isotype control (APC Mouse IgG2a, κIsotype Ctrl (FC) Antibody, Brilliant Violet 421™ Mouse IgG1, κ Isotype Ctrl Antibody, 100 µg/mL, San Diego, CA, USA) were added and the cells were incubated on ice in the dark for 1 h. After addition of 1 mL staining buffer the cells were centrifuged and the supernatant was removed. Cells were resuspended in 500 µL staining buffer and measured using the FACS Canto II (BD Biosciences, Heildelberg, Germany). At least 50,000 events of each sample were recorded. All measurements were executed three times independently.

### 4.6. Statistical Analysis

Results are expressed as mean ± standard deviation of at least three independent measurements. Statistical analysis was performed using unpaired ANOVA and *post-hoc* testing according to Dunnett (SPSS for Windows; SPSS Inc, version 23.0.0.0, Chicago, IL, USA). Statistical significance was set at *p* < 0.05 (in figures marked with an asterisk). ** *p* < 0.01 and *** *p* < 0.001 were indicated, respectively.

## Abbreviations

ASC	Adipose-derived stem cells
HDMEC	Human dermal microvascular endothelial cells
BrdU	5-Bromo-2′-Deoxyuridine
WST-8	(2-(2-methoxy-4-nitrophenyl)-3-(4-nitrophenyl)-5-(2,4-disulfophenyl)-2*H*-tetrazolium)
